# Assessment of Speech Processing and Listening Effort Associated With Speech-on-Speech Masking Using the Visual World Paradigm and Pupillometry

**DOI:** 10.1177/23312165241306091

**Published:** 2025-01-12

**Authors:** Khaled H. A. Abdel-Latif, Thomas Koelewijn, Deniz Başkent, Hartmut Meister

**Affiliations:** 1 Faculty of Medicine and University Hospital Cologne, Department of Otorhinolaryngology, Head and Neck Surgery, 14309 University of Cologne, Cologne, Germany; 2 Jean Uhrmacher Institute for Clinical ENT-Research, 14309 University of Cologne, Cologne, Germany; 3 Department of Otorhinolaryngology/Head and Neck Surgery, University Medical Center Groningen, 3647 University of Groningen, Groningen, The Netherlands; 4 Research School of Behavioural and Cognitive Neurosciences, Graduate School of Medical Sciences, 3647 University of Groningen, Groningen, The Netherlands

**Keywords:** visual world paradigm, speech-on-speech masking, gaze fixation, pupil dilation, listening effort

## Abstract

Speech-on-speech masking is a common and challenging situation in everyday verbal communication. The ability to segregate competing auditory streams is a necessary requirement for focusing attention on the target speech. The Visual World Paradigm (VWP) provides insight into speech processing by capturing gaze fixations on visually presented icons that reflect the speech signal. This study aimed to propose a new VWP to examine the time course of speech segregation when competing sentences are presented and to collect pupil size data as a measure of listening effort. Twelve young normal-hearing participants were presented with competing matrix sentences (structure “name-verb-numeral-adjective-object”) diotically via headphones at four target-to-masker ratios (TMRs), corresponding to intermediate to near perfect speech recognition. The VWP visually presented the number and object words from both the target and masker sentences. Participants were instructed to gaze at the corresponding words of the target sentence without providing verbal responses. The gaze fixations consistently reflected the different TMRs for both number and object words. The slopes of the fixation curves were steeper, and the proportion of target fixations increased with higher TMRs, suggesting more efficient segregation under more favorable conditions. Temporal analysis of pupil data using Bayesian paired sample *t*-tests showed a corresponding reduction in pupil dilation with increasing TMR, indicating reduced listening effort. The results support the conclusion that the proposed VWP and the captured eye movements and pupil dilation are suitable for objective assessment of sentence-based speech-on-speech segregation and the corresponding listening effort.

## Introduction

In daily life, listeners frequently find themselves in environments where they need to focus on a single talker's speech amid other simultaneous conversations. This situation is often referred to as the “cocktail party problem” (Cherry, 1953). Recognizing speech in such environments is notably challenging due to the spectro-temporal overlap of the concurrent talkers. Consequently, certain segments of the target speech may become inaudible. Furthermore, as the masker itself is speech, there is a potential for stimulus uncertainty, leading to possible confusion between the target and competing speech. At a cognitive level speech maskers that carry meaningful content have a stronger impact on the perception of the target speaker than meaningless maskers, such as steady noise. For example, both Koelewijn et al. (2012) as well as Zekveld et al. (2014) showed that their participants exhibited increased mental effort required for speech perception when exposed to a single-talker masker compared to fluctuating noise that had the same spectrotemporal overlap as the single-talker masker. This effect is attributed to factors such as lexical-semantic interference and attentional capture by the masker (Kidd et al., 2007; Mattys et al., 2012).

In order to extract the target, competing speech signals have to be segregated and the target elements have to be streamed, i.e., connected across time (overview in Bronkhorst, 2015). Several cues contribute to the segregation of the competing auditory streams including voice characteristics, spatial cues, and intensity cues. For instance, in collocated speech-on-speech masking scenarios, improvements in speech recognition were observed for higher target-to-masker ratios (TMRs) compared to lower TMRs, which was mainly due to the smaller amount of target-masker confusions (Brungart, 2001).

The established method of assessing speech recognition usually involves speech recall tasks. In these tasks, participants are commonly presented with a target speech, such as single words or sentences, while being exposed to background noise or a speech masker. The participant's task is to verbally repeat as many words from the target speaker as possible or, if a closed-set material is used, to indicate them on a screen. The percentage of correctly repeated words or, in the case of adaptive tests, the TMR linked to a predefined recognition threshold (e.g., 50%) is then used as the outcome measure. Speech recognition tests are widely used in research and also serve as an important clinical tool to evaluate the functional status of the auditory system. However, it should be noted that the results do not necessarily provide in-depth insights into the underlying speech processing. Aspects such as temporal dynamics and cognitive load involved in the process are not captured by the recognition measure.

A promising and increasingly applied tool for gaining deeper insights into the time course of speech processing is the Visual World Paradigm (VWP, Cooper, 1974). In the VWP, auditory stimuli are simultaneously presented with corresponding visual icons. The core principle of the VWP lies in the interrelation between gaze fixations and speech processing, as eye movements continuously evolve during the process of speech comprehension and reflect the listener's focus of attention (Huettig & Altmann, 2005). During the exploration of the visual objects, participants’ eye movements are tracked with a camera. This reveals the extent to which listeners consider different interpretations over time based on the speech cues heard up to that point, which supports the investigation of speech processing dynamics.

The VWP has proven useful in the investigation of speech processing (e.g., Ben-David et al., 2011 Wagner et al., 2016; Wendt et al., 2014). Ben-David et al. (2011) assessed the effects of aging and noise on real-time spoken word recognition. They presented groups of younger and older adults with target nouns in quiet or against background noise. In addition to the target word, similar-sounding phonological competitors were displayed as visual objects in their VWP. Depending on the type of competitor and the background condition, distinct age-related differences emerged, although no such differences were found in recognition accuracy. A study by Wendt et al. (2014) investigated the processing demands associated with different sentence structures, such as subject-verb-object, or object-verb-subject clauses. They observed prolonged sentence processing durations, as evidenced by longer disambiguation-to-decision delays measured by gaze fixations, indicating higher processing demands for linguistically complex clauses. Wagner et al. (2016) presented young normal-hearing listeners with natural and degraded speech and displayed the target word, a phonological competitor, and two unrelated objects in their VWP. The target fixation curves were steeper and the transitions between target and competitor fixation were somewhat earlier in natural speech, suggesting that listeners take longer to make decisions about the lexical target and are less confident when exposed to degraded speech. These examples show that considering eye-gaze measurements may give valuable information about various aspects of speech processing not necessarily captured by speech recall tasks. However, apart from a study by Kaplan et al. (2021), to our knowledge, speech-on-speech masking has not yet been considered.

Speech processing relies on mental operations that can constitute a cognitive load, making the act of listening effortful. The FUEL model (Framework for Understanding Effortful Listening, Pichora-Fuller et al., 2016) defines listening effort as the “deliberate allocation of mental resources to overcome obstacles in goal pursuit when carrying out a task involving listening.” Given that the VWP involves tracking eye gaze, it provides an opportunity to concurrently measure pupil dilation (Van Engen & McLaughlin, 2018). Pupillometry is thought to reflect the cognitive processing load associated with listening effort (Zekveld et al., 2018) and is increasingly used in language and hearing research. It offers a distinct advantage as a time-series measurement, which is crucial for examining rapid auditory encoding and cognitive processing in the comprehension of speech (Winn et al., 2018).

Giuliani et al. (2021) found pupillometry to be the most sensitive measure of listening effort when compared to alternative methods such as the dual-task paradigm, skin conductance response, and self-reported effort. Pupil responses show sensitivity to the demands of speech processing, with greater pupil dilation—indicating increased listening effort—associated with decreasing speech recognition (Zekveld et al., 2018). In speech-on-speech studies, Koelewijn et al. (2012) showed that speech maskers caused a larger pupil dilation than fluctuating noise maskers (with a similar temporal envelope as the speech masker), although speech recognition was similarly high. In another study, young normal-hearing listeners were asked to recall a predefined target sentence while ignoring a simultaneously presented distractor sentence (Koelewijn et al., 2015). The target was defined based on location, which was fixed or random. Pupil responses indicated a higher processing load with random presentation, which was associated with higher stimulus location uncertainty and greater difficulty to focus attention. This result was also confirmed in listeners with mild-to-moderate sensorineural hearing loss (Koelewijn et al., 2017). These studies show that pupillometry might be a sensitive measure for capturing cognitive processing load when competing sentences are presented.

In the present study, we introduce a VWP that specifically addresses sentence-based speech-on-speech masking, combining the measurement of eye gaze as a potential proxy for attention and pupil size as a potential proxy for cognitive load. The method refers to the ability to segregate two competing sentences and to recognize predefined target words. To this end, two words of the target sentence and the corresponding word positions of the masker sentence were displayed in the VWP. Different TMRs were applied and results obtained with a group of young normal-hearing listeners are presented.

To our knowledge, previous research has primarily used the VWP to study the speech processing of single words, which limits our understanding of how speech comprehension evolves over time. Our goal was to track speech processing at two different word positions, offering a more detailed temporal framework. Additionally, we aimed to examine the sensitivity of the VWP in detecting differences between TMR conditions compared to a speech recall task. If the VWP proves effective in differentiating TMRs, it could also serve as a valuable method for research that does not rely on verbal responses, making it particularly beneficial for individuals with impaired speech production.

We hypothesized that, compared to unfavorable TMRs, higher TMRs would enable the listeners to focus on the target sentences, which would be reflected in increased gaze fixations and decreased pupil dilation, as captured by the proposed VWP.

## Methods

### Participants

Twelve normal hearing listeners were recruited, four males and eight females, aged 22–27 years (mean 24.2, SD 1.8). Their pure tone thresholds were ≤25 dB HL across all audiometric frequencies from 125 to 8000 Hz. All participants were native German speakers and had normal or corrected-to-normal vision. Vision accuracy was tested using the HOTV near-chart test at 35.5 cm viewing distance (Simons, 1983). Participants were instructed not to consume any caffeine on the day of the experiment since this can affect pupil dilation (Redondo et al., 2020). Prior to data collection, participants were informed about the study details and written informed consent was obtained. The study protocol was approved by the local ethics committee (protocol number 20-1372). Reimbursement was € 10,-/h. The experiment duration was approximately 1.5 h.

### Stimuli

The Oldenburg sentence test (OLSA, Wagener et al., 1999), a test frequently used in scientific and clinical settings in Germany, was used to assess speech recognition in speech masking. The OLSA is a matrix test presenting sentences composed of five words (name – verb – numeral – adjective – object) and ten possible alternatives for each word position, for example, “Stephen buys seven wet knifes” or “Thomas gives eighteen white cups.” Sentences are syntactically correct but semantically unpredictable, thus allowing repeated testing. Moreover, their fixed structure and low context information make the OLSA highly suitable for assessing the effects of lexical-semantic interference (Rählmann & Meister, 2017). The target sentence was always indicated by the name “Stephen” and the participants’ task was to listen to the target and to ignore the masker sentence. Both the target and the masker sentences were uttered by the same speaker and had the same duration of 2.5 s. The male speaker of the OLSA was used, and the competing sentences were presented at TMRs of 0, 2.5, 4.5, and 6.5 dB, ranging from challenging to easier conditions. The target sentence was always presented at 65 dB SPL. The TMRs were the result of a pilot experiment with the aim of achieving intermediate to near perfect speech recognition. The speech material was presented diotically via headphones (Sennheiser HD 650, Wedemark, Germany).

**Figure 1.**
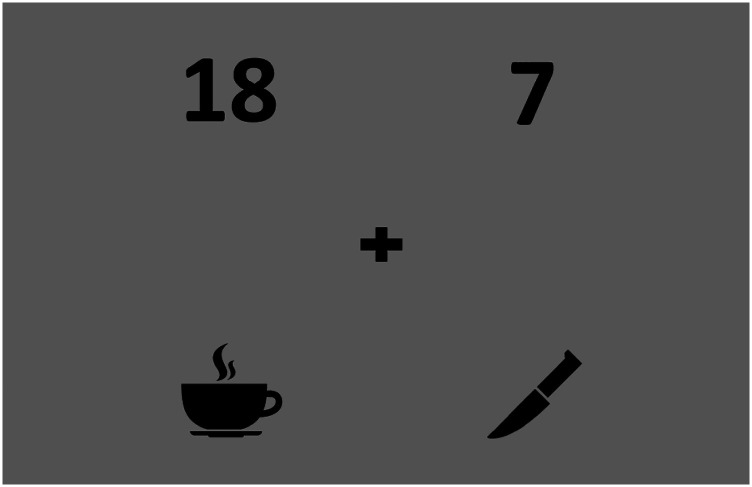
Example of a visual stimulus for target sentence: “Stefan kauft sieben nasse Messer” (Stephen buys seven wet knifes) illustrated by the number “7” and the “knife” icon. Masker sentence: “Thomas gibt achtzehn nasse Tassen” (Thomas gives 18 white cups) illustrated by the number “18” and the “cup” icon. A fixation cross is presented in the center of the screen.

The visual stimuli representing the OLSA comprised 10 unique numbers and 10 unique objects, each consisting of four icons, two numbers, and two object icons related to the target and the masker sentence (Figure 1). There were 28 screen combinations. Across the trials, the position of the numbers and objects was counterbalanced with respect to the target and masker in order to avoid any a priori information on the sentence of interest. Moreover, it was important to avoid a possible bias due to pupil foreshortening effects. Foreshortening occurs when the eye rotates away from the eye-tracking camera, resulting in a change in pupil shape (Hayes & Petrov, 2016; Petersch & Dierkes, 2022; Steinhauer et al., 2022). This effect does not have an influence on the results if there is no systematic difference in gaze position across experimental conditions. An exception from counterbalancing was that the numbers were always displayed above the objects.

The decision to place the numbers at the top and the objects at the bottom was made to reduce complexity and provide a more natural visual flow, as it mimics top-to-bottom reading. Our aim was to present a clearly arranged screen in order to avoid complex visual search processes that could induce additional cognitive load. The luminance of the visual stimuli was balanced. This was tested in a control experiment and was found to be suited in excluding any luminance effects on pupil dilation.

### Eye-Tracking Experiment Apparatus

The EyeLink^®^ 1000 Plus eye-tracker (SR-Research Ltd., Mississauga, Ontario, Canada) was used for the experiment. Eye gaze and pupil diameter were recorded from the right eye at a sampling frequency of 500 Hz using a 25-mm lens (Eyelink High speed for DM-890/AM-890). A chin rest (SR Research Head Support) was used to stabilize the participant's head and they were asked to keep their position fixed during the eye-tracking recordings. The distance from the participant's chin to the desktop camera was 46 cm and a total of 71 cm to the computer monitor (HP EliteDisplay E243, HP Development Company, L.P., w: 53 cm, h: 33 cm). Regarding the placement of the numbers and icons on the screen, the visual angle was 7.22°. The measurements were performed in a sound-treated booth (l: 267 cm, w: 196 cm, h: 268 cm). A dimmed, homogeneous light was provided in the booth, which was constant for all test runs.

### VWP Task Procedure

Prior to the VWP task, participants were familiarized with the OLSA speech material by presenting 40 sentences in quiet. They were then familiarized with the icons of the visual stimuli in paper form. This was followed by training with the combined visual (screen) and auditory stimuli, performed with a list of 40 OLSA sentences. The experiment commenced with calibration and validation of the eye movements. The right eye was always chosen for recording. Different background gray colors were then set based on different RGB values, namely (0,0,0), (26, 26,26), (51,51,51), (77,77,77), (102, 102,102), (128,128,128), (153,153,153), (179,179,179), (204,204,204), (230,230,230), and (255,255,255). This was done to assess the individual dynamic range of the pupil to avoid any ceiling or floor effects. The background grayscale closest to the mean pupil diameter was used for the upcoming trials of the experiment.

**Figure 2.**
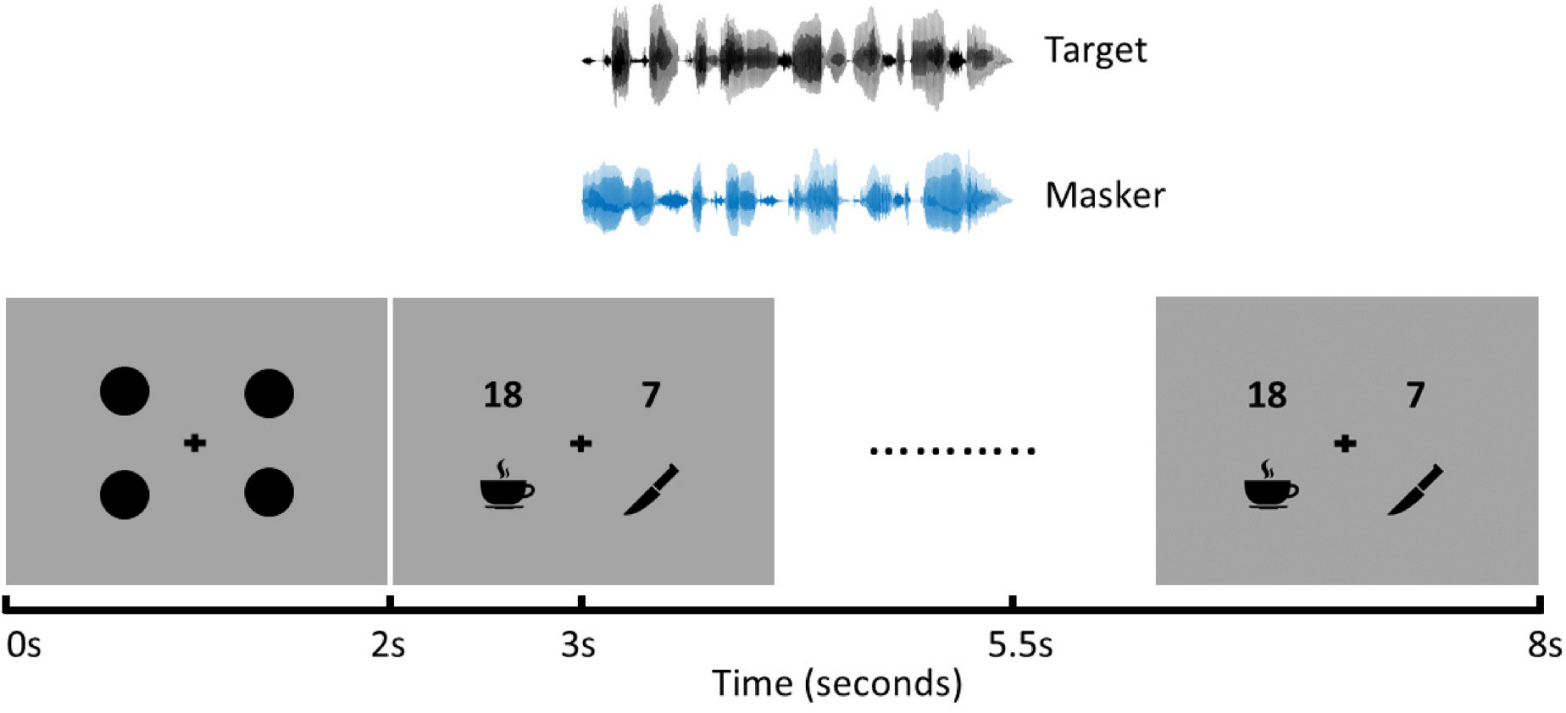
Schematic diagram of the VWP. Initially, the image containing uniform circles is displayed, followed after 2 s by the presentation of the visual stimuli. Subsequently, two auditory streams are introduced one second after the visual stimuli, each lasting for 2.5 s. The visual stimuli persist until the end of the trial (8 s).

In order to avoid early eye movements driven by visual search processes, four uniform circles were displayed before the visual stimuli representing the number and object of the competing sentences (Figure 2). The luminance of the image with the uniform circles corresponded to the mean luminance of all pictures of the VWP. This approach was tested in a control experiment and was found to be suited in excluding any luminance effects on pupil dilation within a trial.

Overall, a trial of the VWP began with the presentation of the uniform circles, followed by the display of the visual stimulus and the subsequent aural presentation of the competing sentences (Figure 2). Participants were instructed to focus their gaze on the cross in the center of the screen, to concentrate on the target sentence, and to look at the number and the object icon belonging to the target as soon as the two competing sentences were presented. The participants were instructed not to respond verbally, since this could affect the pupil data (Winn et al., 2018). Four experimental blocks were conducted, each with seven sentences at four different TMRs, resulting in a total of 112 trials. Presentation of the sentences was pseudo-randomized. The participants went through two consecutive blocks. They then had a break of at least 15 min. Finally, the last two blocks of the VWP were carried out, resulting in a total of 28 trials per TMR.

### Speech Recall Task Procedure

Since the VWP task did not allow participants to verbally respond, no speech recognition data was collected. Hence, in addition to the VWP task, a corresponding speech recall task was carried out with an OLSA list of 40 sentence pairs including the four different TMRs in pseudorandom order. The task was to repeat back as many words as possible from the target speaker. As the name “Stephen” was again used as the keyword this referred to four words per sentence resulting in 40 words per TMR. The speech recall task took about 10 min and was conducted after the break before the last two blocks of the VWP were assessed.

### Analysis

The Experiment Builder software (SR Research Experiment Builder 2.3.1, Mississauga, Ontario, Canada: SR Research Ltd) was used to define a hidden circular Area of Interest (AoI) around each icon within the picture of the VWP. This enabled us to capture all gaze fixations that fell into these AoIs, consistently maintaining the same distance from the fixation cross at the center of the picture. The coordinates of the AoIs were given to match the corresponding icon and were labeled as target number, target object, masker number, and masker object.

In the data analysis phase, gaze fixations were extracted from the Experiment Viewer software (SR Research Ltd., version 3.1) using the time course analysis approach (binning). The binning interval was set to 20 milliseconds. The exported data represented the percentage of total right-eye samples in the current bin that fell within the AoI, relative to the total number of samples.

Additionally, two parameters were computed to assess the proportion of maximum gaze fixations and the slope of the fixation curve. To achieve this, the data of each participant was first smoothed by applying a moving average filter (window size 30 samples). Then, the maximum gaze fixation over the time course was identified and the slope between the 20% and 80% points relative to the maximum was assessed. MATLAB (Version: R2020b, The MathWorks Inc., Natick, Massachusetts, 2020) was used for this analysis.

The maximum gaze fixation and slope recorded during the VWP task were analyzed separately using a repeated-measures analysis of variance (rmANOVA) with TMR and word position (number, object) as within-subject factors. A rmANOVA with TMR as a within-subject factor was also conducted for the results of the speech recall task. If the assumption of sphericity was violated, Greenhouse–Geisser corrections were applied. Paired samples *t*-tests with Bonferroni correction were conducted as post hoc analyses. Analyses were performed using IBM SPSS Statistics v. 28.

For pupil data preprocessing and analysis, the CHAP software (Hershman et al., 2019) was used, which included linear interpolation for blink reconstruction. Following preprocessing, we calculated the relative pupil dilation change from baseline using the divisive baseline correction equation (Hershman et al., 2019). The baseline was defined as the average 500 ms before the onset of the aurally presented competing sentences. Pupillometry data was analyzed by means of Bayesian temporal analysis using CHAP, incorporating a paired-sample *t*-test (Cauchy prior width of *r* = .707) to measure effect size, assuming differences between conditions. Holm–Bonferroni correction was applied by the CHAP software. The Bayes factor BF10 (Jeffreys, 1961) was taken as an index for contrasting the different TRM conditions, comparing the likelihood of the alternative hypothesis (indicating a difference between conditions) against the null hypothesis (indicating no difference). For example, a BF10 of 3–10 suggests moderate evidence favoring the alternative hypothesis over the null hypothesis, and a BF10 larger than 10 suggests strong evidence (Lee & Wagenmakers, 2014).

## Results

### Speech Recall Task

**Figure 3.**
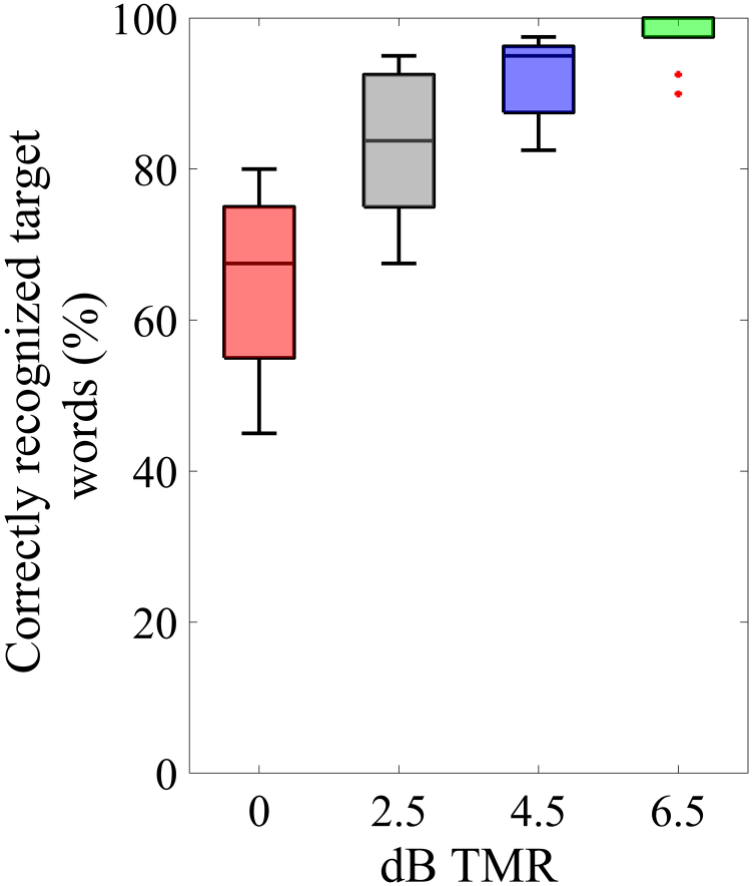
Results of the speech recall task. The median percentage of correctly recognized target words is shown. The colors are referring to the different TMRs. Boxes depict 25% and 75% percentiles. The whiskers extend to the most extreme data points not considered outliers, and the outliers are plotted individually using a red dot marker symbol.

The individual performance in the speech recall task was assessed for the four different target-to-masker ratios. Depending on the TMR, performance ranged from intermediate to near-perfect speech recognition. Figure 3 shows the percentage of correctly repeated words out of four possible target words for each condition. A rmANOVA was conducted with TMR as a within-subject factor. Mauchly's test indicated that the assumption of sphericity was violated (*χ*²(5) = 19.53, *p* = .002); therefore, Greenhouse–Geisser corrected tests are reported (*ε* = .59). The results revealed a significant main effect of TMR condition (*F*(1.7, 19.5) = 42.25, *p* < .001, *ηp*² = .793). Planned comparisons with Bonferroni correction revealed significant differences in word recognition for most TMR conditions. Specifically, recognition at 6.5 dB TMR was significantly higher compared to both 2.5 dB (*p* < .001) and 0 dB (*p* < .001). However, there was no significant difference between 6.5 dB and 4.5 dB (*p* = .072). Additionally, a significant difference was noted between 4.5 dB and 0 dB (*p* < .001), whereas no significant difference was observed between 4.5 dB and 2.5 dB (*p* = .078). Furthermore, word recognition in the 0 dB condition was significantly lower than in the 2.5 dB condition (*p* < .001). Please refer to the Appendix for detailed information.

### VWP Task—Eye Gaze Fixations

**Figure 4.**
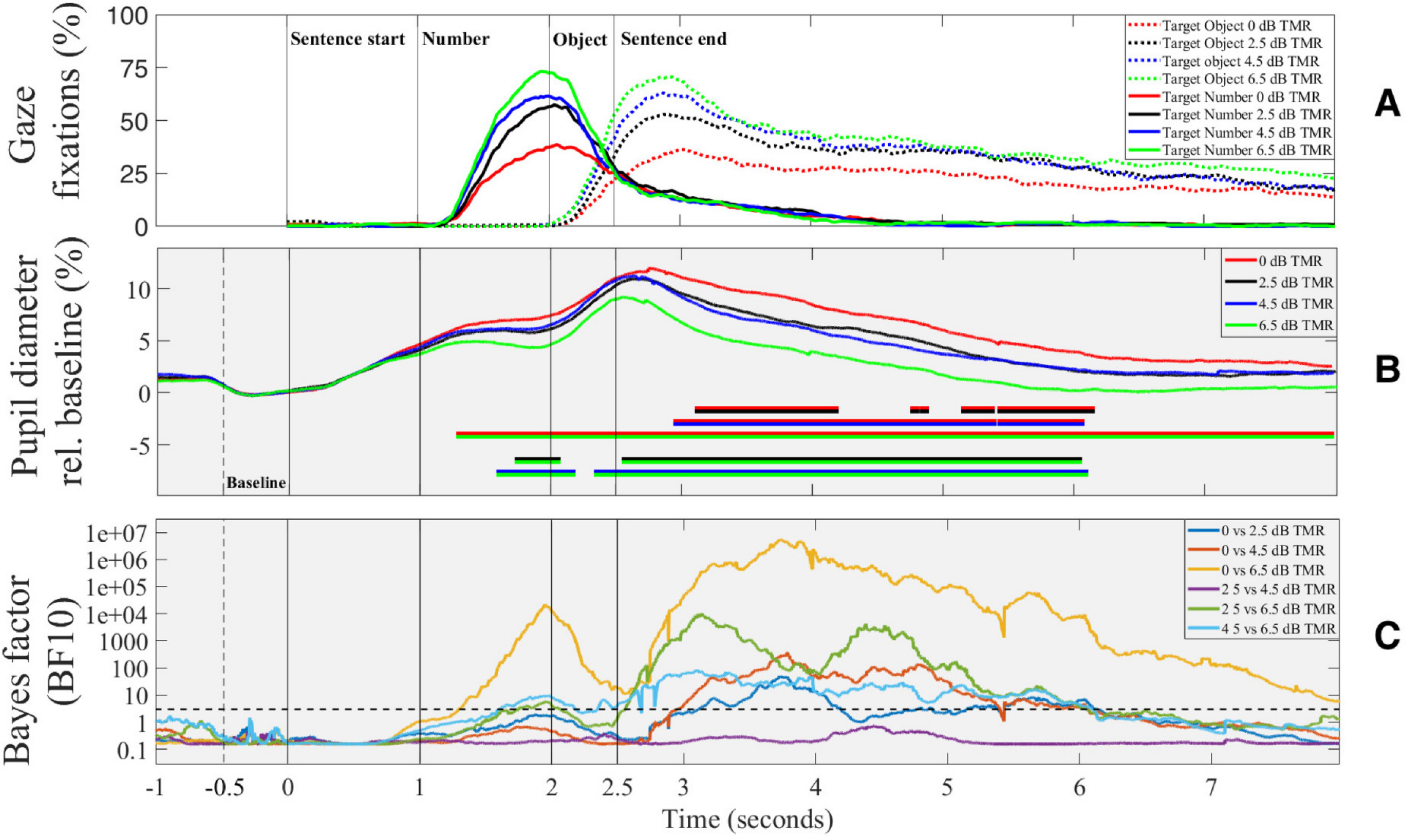
Results of the VWP. (a) Gaze fixations in percentage over time. Colors indicate the different TMRs. (b) Mean relative pupil diameter compared to the baseline (interval 500 ms before sentence onset). Horizontal lines in the lower part of the figure support evidence of differences between conditions (Temporal Bayesian analysis of pupil response, Hershman et al., 2023), based on the Bayes factor BF10. (c) Bayes factor BF10 as a function of time for each comparison between conditions of the pupil response. The dashed horizontal line indicates a BF10 of 3.

**Figure 5.**
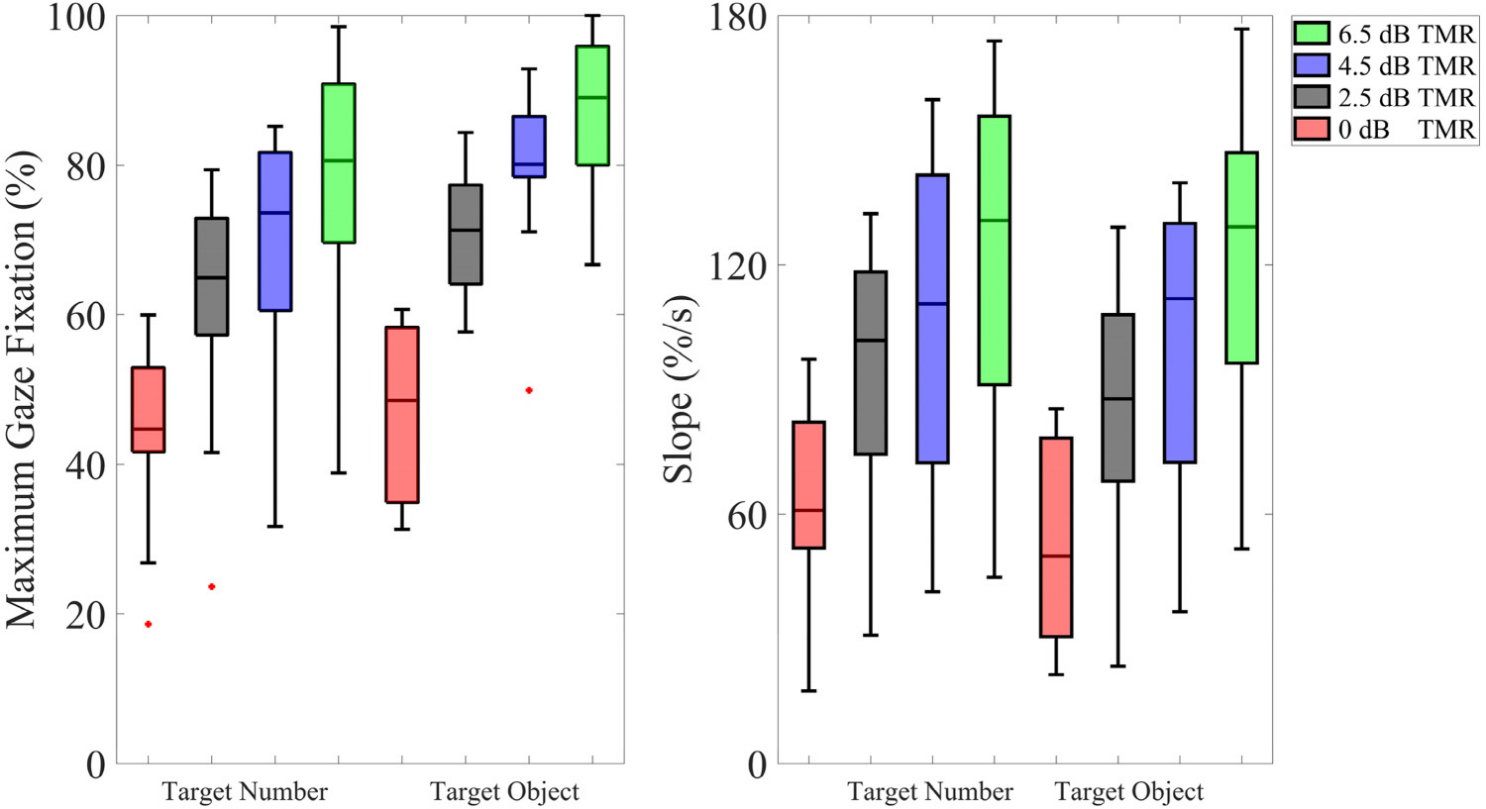
Median of maximum gaze fixations (left) and the slope of the fixation curve (right) for target number and target object. TMR (6.5, 4.5, 2.5, 0) dB are color coded with green, blue, black, red, respectively. Boxes depict 25% and 75% percentiles. The whiskers extend to the most extreme data points not considered outliers, and the outliers are plotted individually using a red dot marker symbol.

The proportion of gaze fixations over time for the target number and the target object at different TMRs is shown in Figure 4(a). Lower TMRs are associated with lower maximum and shallower fixation curves. Figure 5 presents the results for the two factors analyzed, namely the maximum gaze fixation value and slope, for both the target number and the target object. As anticipated, higher TMRs are associated with a higher proportion of maximum gaze fixations and a steeper slope.

A rmANOVA for the maximum gaze fixation, with TMR condition and word position (number/object) as the within-subjects variables, was conducted. Mauchly's test indicated that the assumption of sphericity was violated for TMR (χ²(5) = 14.41, *p* = .014); therefore, Greenhouse–Geisser corrected tests are reported (ε = .54). The results revealed a significant main effect of TMR (*F*(1.6, 17.8) = 150.47, *p* < .001, *ηp*² = .932) and word position (*F*(1, 11) = 6.383, *p* = .028, *ηp*² = .367). There was also a significant interaction between TMR and word position (*F*(3, 33) = 3.51, *p* = .026, *ηp*² = .242).

Post hoc pairwise comparisons with Bonferroni correction for the different TMRs revealed that the maximum gaze fixation was significantly higher for 6.5 dB compared to 0 dB (*p* < .001), 2.5 dB (*p* < .001), and 4.5 dB (*p* < .001). Additionally, the maximum gaze fixation was significantly higher for 4.5 dB than 2.5 dB (*p* < .001) and 0 dB (*p* < .001) as well as for 2.5 dB than 0 dB (*p* < .001). Concerning the interaction between word position and TMR, a significant gaze difference in word position was observed for the two highest TMRs, while no significant difference was found for the two lowest TMRs (please refer to the Appendix for detailed information).

Similarly, a rmANOVA for the slope of the fixation curve, with TMR condition and word position as the within-subjects variables, was conducted. Mauchly's test indicated that the assumption of sphericity was violated for TMR (*χ*²(5) = 17.05, *p* = .005); therefore, Greenhouse-Geisser corrected tests are reported (*ε* = .507). The analysis revealed a significant main effect of TMR (*F*(1.5, 16.7) = 74.716, *p* < .001, *ηp*² = .872), with no significant effect observed for word position and no interaction between word position and TMR.

To further investigate whether the slope could differentiate the TMRs, we conducted planned comparisons with Bonferroni correction for TMR. These indicated that the slope was significantly steeper for 6.5 dB compared to 0 dB (*p* < .001), 2.5 dB (*p* < .001), and 4.5 dB (*p* < .001). Additionally, the slope was significantly steeper for 4.5 dB compared to 2.5 dB (*p* = .018), as well as between 4.5 dB and 0 dB (*p* < .001), and between 2.5 dB and 0 dB (*p* < .001).

### VWP Task—Pupil Dilation

The mean pupil diameter as a percentage of the baseline (interval 500 ms before sentence start) is plotted over time in Figure 4(b). As a sign of cognitive load, the pupil dilated in all TMRs compared to the baseline once the sentence started. Pupil size increased further when number and object were presented. The trajectories of the different TMRs began to separate as soon as the number was presented. The pupil size reflected the different TMR values and showed a greater dilation for the more difficult conditions. A Bayes factor (BF10) between 3 and 10 is considered moderate evidence that differences between conditions are three times more likely than the absence of differences, a BF10 between 10 and 100 is regarded as strong evidence. In Figure 4(c) the Bayes factor is plotted as a function of time for each TMR comparison. A BF10 of 3 is shown as a horizontal dashed line. The figure reveals that the strength of BF10 for pupil dilation varied based on the TMR comparison as well as on the time course of processing the individual target words of the sentence.

## Discussion

Our research aimed to explore the potential of using a VWP to assess speech processing in a sentence-based speech-on-speech masking scenario and distinguish listening effort across varying levels of TMR. We hypothesized that higher TMRs would facilitate listeners’ ability to focus on target speech, leading to observable differences in gaze patterns as a measure for attention and pupil dilation as a measure of listening effort.

### Speech Recall Task

The aim was to cover a wide range of speech recognition levels, from moderate to near perfect, but without making recognition too difficult, as this could reduce the motivation to perform the task, with possible consequences for gaze and pupil size (Zekveld et al., 2018). The results of the speech recall task showed that this was feasible by presenting TMRs between 0 and 6.5 dB. Statistical analysis revealed that speech recognition scores differed significantly between all four TMRs except between 4.5 and 2.5 and between 4.5 and 6.5 dB TMR.

### VWP Task—Eye Gaze Fixations

Eye-tracking yielded time-series measurements of fixations during the presentation of the sentences and the corresponding visual icons within the VWP. Starting from a fixation cross in the center of the screen, the participants directed their gaze to the number and the object of the target sentence, as instructed and promoted by the close link between gaze and speech processing. The typical pattern revealed two distinct maxima, one for the target number and one for the target object. On a temporal basis, changes in gaze could be observed at about 200 ms after the presentation of the corresponding word. This delay was in line with expectations, given that adults typically require around 200 to 250 milliseconds to make a saccade, as documented in the research of Minor et al. (2022).

Importantly, the gaze pattern clearly depended on the TMR presented, with higher maximum and steeper slope of the fixation curve for the more favorable TMRs. This was confirmed by the statistical analyses, which showed significantly different maxima for all TMRs and significantly different slopes for all TMRs. The gaze fixations appear to be a sensitive measure to differentiate between TMRs that are near-ceiling conditions, i.e., between 4.5 and 6.5 dB TMR, corresponding to a mean recognition of 92.5% and 98.1%, respectively, which was not statistically significant in the speech recall task.

The maximum gaze reflected the amount of gaze fixations on the corresponding icon and may thus be interpreted as a proxy for the ability to focus attention on the target talker. The analysis of the maxima revealed a significant interaction between word position and TMR. This finding suggests that word position influences attention per TMR, as reflected by the greater variability and lower mean of the target number in comparison to the target object (Figure 5). Specifically, focusing attention on the target appears to be favored in the later word position, at least in the more favorable TMRs. Participants might have been able to track the target object more seamlessly, possibly due to its alignment with the flow of the target sentence. Conversely, participants showed relatively more uncertainty in the case of the target number, potentially indicating a less straightforward segregation between the target and the masker sentence. An alternative explanation could be that participants quickly focused on the target object after the presentation of the number word, which may have reduced the amount of gaze fixations on the number. However, this idea would explain the significant main effect of word position but not necessarily the interaction with the TMR.

At the most favorable TMR of 6.5 dB, about half of the individual maximum gaze fixations for the target number and object were around 90–100%, suggesting that the listeners had no problems directing their attention to the target sentence. At the lowest TMR of 0 dB, however, most fixations on the target words showed a value of around 50%, with a mean value of about 45%. When analyzing the *masker* fixations, the mean value was around 35%, indicating that attention was misdirected to some degree. As TMR increased, the amount of masker fixations approached zero.

Although maximum gaze fixation value and slope are not entirely independent, the latter may give additional temporal information. The slopes for the more favorable TMRs were steeper, suggesting that the listeners were more quickly able to identify the target when presented against the competing sentence. The increase in processing time for the harder TMR conditions may reflect the increased cognitive processing costs, consistent with analogous findings in previous studies (Wendt et al., 2014).

### VWP Task—Pupil Dilation

In terms of the modification of the TRM, pupil size clearly reflected the differing demands of separating the competing sentences and focusing the attention on the target. By means of Bayesian statistics, i.e., the Bayes factor BF10, it could be shown that most of the TMR-related pupil dilations differed significantly from each other. Predictably, this was most evident for the comparison of 0 dB TMR with 6.5 dB yielding BF values up to 1 E + 6 over the duration of about 1.2 s after stimulus onset until the end of the sequence. Significant differences were also found for the other TMR comparisons, especially in the temporal range of the presentation of the number and object word and after the offset of the stimulus, except between TMRs of 2.5 and 4.5 dB.

Basically, the pupil started to dilate approximately 500 ms after the onset of the competing sentences, compared to the baseline. This pattern is consistent with the observations of Winn et al. (2018) and indicates the cognitive load associated with processing the speech streams. Distinct maxima could be observed for the target number and the target word, and after sentence presentation, pupil dilation persisted but slowly returned to the magnitude of baseline values. This pattern might reflect the fact that pupil size mirrors a complex and potentially interrelated mixture of autonomous nervous system contributions (Zekveld et al., 2018): First, a general cognitive load with the processing of speech might be observed, which is particularly pronounced for speech maskers (Koelewijn et al., 2012; Wendt et al., 2018; Zekveld et al., 2014). Secondly, additional effects of the attentional focus (i.e., on the two words presented within the VWP) and thirdly, memory processing after the stimulus had been presented, could play a role (Zekveld et al., 2018). However, the prolonged pupil dilation after stimulus offset could not be regarded as a response preparation, as no verbal response was elicited from the participants. However, it is plausible that this dilation signifies the effort required to sustain intentional attention focused on the target object, supported by the continuous fixation of gaze on the corresponding icon.

Finally, since we combined eye-tracking and pupillometry, eye movements could have an effect, as McGinley et al. (2015) have shown a possible influence of locomotion on pupil dilatation. We followed the specific recommendations addressing the combination of eye-tracking with pupillometry given in Winn et al. (2018). However, even if eye movements had influenced pupil size to some extent, this influence would have been evenly distributed across the different conditions due to the balanced study design, rendering a strong impact on the parameter of interest (i.e., TMR) unlikely. As for the effect of pupil foreshortening, according to Steinhauer et al. (2022), the impact is minimal because the visual angle was less than 10°.

### General Discussion

A general advantage of the proposed visual word paradigm is the simultaneous assessment of eye gaze and pupil size. Several studies, including those conducted by Klingner (2010), Wagner et al. (2016), Ayasse et al. (2017), and Kaplan et al. (2021), have combined the use of VWP and pupillometry.

To our knowledge, Kaplan et al. (2021) were the only ones to explore the application of these two measures to examine speech-on-speech masking. They presented meaningful target sentences with a specific keyword that was considered as a visual object in the VWP. Additionally, a phonological competitor and two unrelated distractors were presented visually. Target sentences were presented either in quiet or against two-talker maskers at either 0 or −5 dB TMR. It was found that participants fixated less on the target icon with more intense masking and that pupillary responses increased with the poorer TMR, aligning with the findings of our study.

In our study, several methodological modifications were introduced compared to previous studies. First, the inclusion of two visual target icons during speech-on-speech masking deviated from the typical VWP experiment, which usually includes only one target icon. This adaptation allowed us to assess speech segregation at two distinct time points within the sentence, which facilitated the exploration of the temporal dynamics in speech processing. Moreover, a single-talker masker presenting sentences of the same type as the target was considered, providing a direct competitor to each of the target words.

Secondly, manual (e.g., by mouse-click) or verbal responses were not given in the VWP, which facilitated a more precise examination of pupil dilation by eliminating potential influences from behavioral responses, which could have complicated the assessment and interpretation of the data (Visentin et al., 2022; Winn et al., 2018). As a consequence, the analysis encompassed the entire time course over a trial, facilitating the observation of the pupil's response from baseline until its return back to baseline after the sentence's conclusion. This analysis showed that the outcome of the VWP closely reflected the word recognition scores acquired with the speech recall task and that it was even sensitive to changes in the TMR, where recognition was close to the ceiling. Hence, it may be suitable for examining speech-on-speech masking when verbal responses cannot or should not be given by the listeners. Thus, this approach may be particularly suitable for studying speech processing in certain cases using fMRI, where spoken responses can introduce noise (Birn et al., 1998; Volfart et al., 2024). Using eye movements as a response method makes it also suitable for individuals with speech impairments, such as those with Parkinson's disease, where speech production may be limited. If eye movements remain functional, gaze can be used to assess language processing in this population (Aveni et al., 2023).

### Limitations and Future Directions

This study examined sentence-based speech-on-speech masking using a VWP that provides time-series measurements of eye gaze fixations and pupil size. As a first step, this paradigm was assessed in young normal-hearing participants when the TMR changed between the competing sentences. While the small sample size of 12 NH listeners limits the statistical power, it was appropriate given the simplicity of our design, with TMR as the only independent variable and a homogenous participant group. Future research should expand these findings to larger sample sizes, particularly in more heterogeneous populations with varying hearing abilities or in studies with multiple independent variables.

It is unclear what results would have been obtained with other listener groups. For example, Wendt et al. (2015) found that adult hearing-impaired listeners exhibited prolonged processing durations compared to individuals with NH listeners, as evidenced by their eye gaze fixations in a VWP, despite both groups having similar levels of speech recognition. In contrast, Ayasse et al. (2017) showed that older participants with hearing loss did not exhibit slower speech processing compared to young normal-hearing individuals, as observed by eye gaze measurements. However, the study also revealed that the older hearing impaired participants exhibited additional cognitive processing effort, as evidenced by their greater pupil dilations compared to their counterparts with NH.

In addition to the TMR modified in the present study, other factors such as spatial or voice cues are also important for the segregation of competing speech signals. Here, cochlear implant (CI) users are of particular interest due to their restricted access to spectrotemporal cues (e.g., El Boghdady et al., 2019; Meister et al., 2020). These studies have shown large interindividual variability in speech recognition with speech maskers. Increased variability is also expected in eye gazing and pupillometry measurements among CI users (Blamey et al., 2013; Wagner et al., 2019). The investigation of different listener groups and the consideration of additional cues for talker segregation will thus be the focus of future use of the proposed VWP.

## Appendix

**Table:**

*Pairwise comparisons*						
Percentage of correctly recognized words: MEASURE_1						
(*I*) Factor 1	(*J*) Factor 1	Mean difference (*I* − *J*)	Std. error	Sig.^b^	95% Confidence interval for difference^b^	
					Lower bound	Upper bound
0 dB	2.5 dB	−17.500^a^	3.168	.001	−27.664	−7.336
	4.5 dB	−27.292^a^	4.475	<.001	−41.648	−12.936
	6.5 dB	−32.917^a^	2.868	<.001	−42.116	−23.717
2.5 dB	0 dB	17.500^a^	3.168	.001	7.336	27.664
	4.5 dB	−9.792	3.307	.078	−20.400	.816
	6.5 dB	−15.417^a^	2.534	<.001	−23.548	−7.286
4.5 dB	0 dB	27.292^a^	4.475	<.001	12.936	41.648
	2.5 dB	9.792	3.307	.078	−.816	20.400
	6.5 dB	−5.625	1.875	.072	−11.640	.390
6.5 dB	0 dB	32.917^a^	2.868	<.001	23.717	42.116
	2.5 dB	15.417^a^	2.534	<.001	7.286	23.548
	4.5 dB	5.625	1.875	.072	−.390	11.640

*Pairwise comparisons*						
Maximum gaze fixation: MEASURE_1						
(*I*) TMR	(*J*) TMR	Mean difference (*I* − *J*)	Std. error	Sig.^b^	95% Confidence interval for difference^b^	
					Lower bound	Upper bound
6.5 dB	4.5 dB	.082^a^	.015	<.001	.035	.129
	2.5 dB	.157^a^	.021	<.001	.090	.225
	0 dB	.363^a^	.025	<.001	.281	.444
4.5 dB	6.4 dB	−.082^a^	.015	<.001	−.129	−.035
	2.5 dB	.075^a^	.012	<.001	.038	.113
	0 dB	.281^a^	.018	<.001	.222	.339
2.5 dB	6.4 dB	−.157^a^	.021	<.001	−.225	−.090
	4.5 dB	−.075^a^	.012	<.001	−.113	−.038
	0 dB	.205^a^	.012	<.001	.167	.244
0 dB	6.4 dB	−.363^a^	.025	<.001	−.444	−.281
	4.5 dB	−.281^a^	.018	<.001	−.339	−.222
	2.5 dB	−.205^a^	.012	<.001	−.244	−.167

**Table:**

*Pairwise comparisons*							
Maximum gaze fixation: MEASURE_1							
TMR	(*I*) Word position	(*J*) Word position	Mean difference (*I* − *J*)	Std. error	Sig.^b^	95% Confidence interval for difference^b^	
						Lower bound	Upper bound
6.5 dB	Target number	Target object	−.098^a^	.034	.016	−.173	−.022
	Target object	Target number	.098^a^	.034	.016	.022	.173
4.5 dB	Target number	Target object	−.114^a^	.037	.010	−.195	−.033
	Target object	Target number	.114^a^	.037	.010	.033	.195
2.5 dB	Target number	Target object	−.089	.041	.054	−.180	.002
	Target object	Target number	.089	.041	.054	−.002	.180
0 dB	Target number	Target object	−.030	.034	.399	−.106	.045
	Target object	Target number	.030	.034	.399	−.045	.106

*Pairwise comparisons*						
Slope: MEASURE_1						
(*I*) TMR	(*J*) TMR	Mean difference ( *I* − *J* )	Std. error	Sig.^b^	95% Confidence interval for difference^b^	
					Lower bound	Upper bound
6.5 dB	4.5 dB	.178^a^	.028	<.001	.088	.269
	2.5 dB	.307^a^	.048	<.001	.152	.461
	0 dB	.636^a^	.061	<.001	.440	.832
4.5 dB	6.5 dB	−.178^a^	.028	<.001	−.269	−.088
	2.5 dB	.128^a^	.034	.018	.020	.237
	0 dB	.458^a^	.052	<.001	.291	.625
2.5 dB	6.5 dB	−.307^a^	.048	<.001	−.461	−.152
	4.5 dB	−.128^a^	.034	.018	−.237	−.020
	0 dB	.329^a^	.029	<.001	.236	.422
0 dB	6.5 dB	−.636^a^	.061	<.001	−.832	−.440
	4.5 dB	−.458^a^	.052	<.001	−.625	−.291
	2.5 dB	−.329^a^	.029	<.001	−.422	−.236

Based on estimated marginal means.

^a^The mean difference is significant at the .05 level.

^b^Adjustment for multiple comparisons: Bonferroni.
